# Population Growth of Soybean Aphid, *Aphis glycines*, Under Varying Levels of Predator Exclusion

**DOI:** 10.1673/031.010.14104

**Published:** 2010-09-10

**Authors:** Lisa N. Meihls, Thomas L. Clark, Wayne C. Bailey, Mark R. Ellersieck

**Affiliations:** ^1^ Department of Entomology, 1–31 Agriculture Bldg., University of Missouri, Columbia, MO 65211; ^2^ Monsanto Company, 700 Chesterfield Pkwy W., Chesterfield MO, 63017; ^3^ 108 Waters Hall, University of Missouri, Columbia, MO 65211; ^4^ Agricultural Experiment Station Statistician, 307E Middlebush Hall, University of Missouri, Columbia, MO 65211

**Keywords:** Predator complex, Predator exclusion, *Orius insidiosus*

## Abstract

Although soybean aphid, *Aphis glycines* Matsumura (Hemiptera: Aphididae), has caused economic damage in several Midwestern states, growers in Missouri have experienced relatively minor damage. To evaluate whether existing predatory insect populations are capable of suppressing or preventing soybean aphid population growth or establishment in Missouri, a predator exclusion study was conducted to gauge the efficacy of predator populations. Three levels of predator exclusion were used; one that excluded all insects (small mesh), one that excluded insects larger than thrips (medium mesh), and one that excluded insects larger than *Orius insidiosus* (Say) (Hemiptera: Anthocoridae), a principal predator (large mesh). Along with manipulating predator exposure, timing of aphid arrival (infestation) was manipulated. Three infestation times were studied; vegetative (V5), beginning bloom (R1), and beginning pod set (R3). Timing of aphid and predator arrival in a soybean field may affect the soybean aphid's ability to establish and begin reproducing. Cages infested at V5 and with complete predator exclusion reached economic threshold within two weeks, while cages with predators reached economic threshold in four and a half weeks. Cages infested at R1 with complete predator exclusion reached economic threshold within five weeks; cages with predators reached economic threshold within six weeks. Cages infested at R3 never reached threshold (with or without predators). The predator population in Missouri seems robust, capable of depressing the growth of soybean aphid populations once established, and even preventing establishment when the aphid arrived late in the field.

## Introduction

The soybean aphid, *Aphis glycines* Matsumura (Hemiptera: Aphididae), was first discovered in the United States in 2000 and has spread throughout the soybean, *Glycine max* L. (Fabales: Fabaceae), growing regions of the North Central United States (Venette and Ragsdale 2004). By 2004, soybean aphid was present in 21 states and two Canadian provinces, encompassing 80% of the soybean production area in North America. The economic threshold of the soybean aphid was estimated to be 273 aphids per plant, assuming a 7 day lead time to reach the economic injury level (674 aphids per plant) (Ragsdale et al. 2007). The soybean aphid has caused significant yield losses in northern soybean-producing states including Illinois (NSRL 2001), Iowa (Rice et al. 2004), Michigan (DiFonzo and Hines 2002) and Minnesota (MacRae and Glogoza 2005).

Observations from Asia indicate that soybean aphid populations were extremely low in environments similar to the North Central United States (Fox et al. 2004). The soybean aphid populations in Asia are believed to be under the control of a number of natural enemies (Van Den Berg et al. 1997; Rongcai et al. 1994; Miao et al. 2007; Han 1997; Liu et al. 2004; Chang et al. 1994; Ma et al. 1986). In China, Wang and Ba (1998) identified coccinellids as principle to soybean aphid suppression due to high predation rates and high populations.

Studies conducted in the Midwest identified key predators of the soybean aphid; these included the insidious flower bug, *Orius insidiosus* Say (Hemiptera: Anthocoridae), and the multicolored Asian lady beetle, *Harmonia axyridis* (Pallas) (Coleoptera: Coccinellidae), which can account for over 85% of all predators in some environments (Rutledge et al. 2004; Fox et al. 2004). Harwood et al. (2007) found little intraguild predation between *O. insidiosus* and *H. axyridis.* The presence of predatory insects may prevent soybean aphid population growth and also reduce established populations (Van Den Berg et al. 1997; Brown et al. 2003; Fox et al. 2004; Rutledge and O'Neil 2005; Costamagna and Landis 2006;). Predatory insects that respond early in the season, and in large numbers, may be more successful in this regard (Fox et al. 2005; Brosius et al. 2007; Yoo and O'Neil 2009). In some Midwest states, ambient levels of predatory insects are capable of controlling soybean aphid populations (Costamagna et al. 2007a). *Orius insidiosus* is the most common predaceous insect in Missouri soybean (Barry 1973; Marston et al. 1979) and may be responsible for suppressing soybean aphid populations below economic levels.

Soybean thrips, *Neohydatothrips variabilis* (Beach) (Thysanoptera: Thripidae), are an important food source for *O. insidiosus* along with the soybean aphid (Harwood et al. 2007; Butler and O'Neil 2008). Before the arrival of the soybean aphid, it was generally accepted that the soybean thrips was the primary prey species of *O. insidiosus* (Marston et al. 1979). Thrips arrive early in the season (unifoliate stage, VI) in both early and late planted soybean, reproduce rapidly, and are abundant by the time *O. insidiosus* arrives (V5–V8 for May planted; V2–V4 for June planted) (Isenhour and Marston 1981b). This relationship may change with the introduction of the soybean aphid. The soybean aphid is an adequate prey item for *O. insidiosus*, and a combination of soybean aphid and thrips resulted in increased survival, development, and fecundity of *O. insidiosus* versus thrips alone (Butler and O'Neil 2007a; Butler and O'Neil 2007b). However, the presence of thrips has been shown to decrease the predation of *O. insidiosus* on soybean aphid (Desneux and O'Neil 2008).

Along with predation, plant properties affect soybean aphid populations (i.e. bottom-up control of aphid numbers). Potassium deficient soybeans have higher soybean aphid populations, possibly due to an increase in free nitrogen in plant phloem or a change in the composition of amino acids in the phloem (Myers and Gratton 2006; Walter and DiFonzo 2007). Plant phenology may also significantly impact soybean aphid population growth, as was seen with *Myzus persicae* and *Aphis fabae* (Williams et al. 1999; Van Den Berg et al. 1997; Kift et al. 1998; Costamagna et al. 2007b).

The exclusion of predators by physical barriers, followed by observations of the prey population, is a method commonly used to assess the importance of predators on a population (i.e. top-down control of aphid numbers) (Luck et al. 1988). Several exclusion studies have been conducted to evaluate the role of predators in the establishment and spread of soybean aphid (Van Den Berg et al. 1997; Liu et al. 2004; Fox et al. 2004; Fox et al. 2005; Desneux et al. 2006; Costamagna and Landis 2006; Miao et al. 2007; Gardiner and Landis 2007; Costamagna et al. 2008; Chacón et al. 2008). All of these studies indicated that predators play a role in suppression of soybean aphid populations. Whenever resident predators are capable of suppressing soybean aphid populations below threshold, insecticide applications can be avoided.

Despite the presence of soybean aphid in southern soybean producing states such as Missouri, yield losses have been limited. Some speculate that soybean aphid rarely reaches economic threshold in Missouri because high summer temperatures negatively affect aphid development. However, this speculation was not supported by preliminary research, as soybean aphid reached outbreak levels in exclusion cages in central Missouri during the summers of 2001 and 2002. Within a three-week period, soybean aphid populations increased from 5–10 per plant to more than 5,000 per plant (T.L.C., unpublished data). These data suggest that temperature was not the primary reason populations remain low in Missouri. It is more likely that resident predators are responsible, as ambient levels of predatory insects are capable of controlling soybean aphids in some Midwestern states (Costamagna et al. 2007a). The purpose of this research was to evaluate the predator complex inhabiting central Missouri soybean fields and to determine their impact on soybean aphid populations at different plant growth stages. This design encompasses top-down (predator exclusion) and bottom-up (plant phenology, i.e. nutritional quality) factors affecting soybean aphid populations.

## Materials and Methods

### Experimental Design

**Figure 1.**
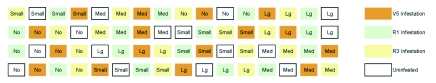
Experimental setup. Cages were sampled at ∼7 day intervals. High quality figures are available online.

The study was conducted at the University of Missouri, South Farms, in the summer of 2004. South Farms (92° 17′ W, 92° 12′ N; elevation ≈ 272 m) is located approximately 5.8 km southeast of University of Missouri campus. Cages were 1.5 m apart and replications were 6 m apart within the soybean field. Fields were cultivated using reduced primary tillage (disc), cages were placed and soybean variety DKB 38–52 (Asgrow® Roundup Ready®, Monsanto Company, www.monsanto.com) was planted six seeds to a cage on 22 June 2004. A non-standard planting density was utilized to facilitate sampling by observers. Cages and nearby plots were kept weed free by the application of Roundup WeatherMAX® (glyphosate) at a rate of 864 g (AI)/ha (Monsanto) on 17 July and 13 August. The experiment was set up as a randomized complete block split-plot design in a 4 × 3 (infestation date × mesh size) factorial arrangement replicated four times, with the main plot of mesh, and a subplot of infestation date (Figure 1). A no mesh treatment was included as a control; however, due to herbivory this treatment was dropped from the analyses. In addition, cages were sampled over time requiring a repeated measures analysis.

### Predator Exclusion Trials

**Figure 2.**
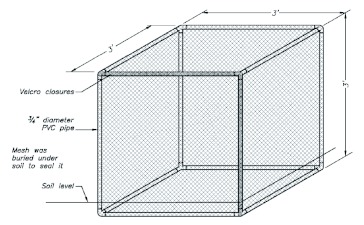
Design of exclusion cages in 2004. Figure by Kelly Schweikert. High quality figures are available online.

Aphidophagous predators (Coccinellidae, Syrphidae, Chrysopidae, and Anthocoridae) and soybean aphid densities were monitored throughout the season. Cage frames were constructed of PVC pipe and fittings (1.3 cm outside diameter; Lasco Fittings, Inc., www.lascofittings.com). Cages were 1 m^3^ with approximately 10 cm placed in the soil and secured with 10 cm wire landscape staples (Figure 2). Three sizes of mesh were used: Econet S (300 squares per cm), Econet L (140 squares per cm) (LS Climate Control Pty Ltd., www.svensson.com.au) and mosquito netting (6 squares per cm) (Econet Specifications http://insect-screen.usgr.com/econet-insectscreen.html). Mesh was sewn to fit the cage frame with excess material on the bottom to allow burial. Mesh was buried in the soil and secured with 10 cm wire landscape staples. Access was provided by Velero® closures along the top and side of one panel.

Mesh sizes were chosen based on predator size. Small mesh (Econet S) was selected to exclude all arthropods, even mites. Medium mesh (Econet L) was selected to exclude all insects larger than thrips and whiteflies. Large mesh (mosquito netting) was selected to exclude all insects larger than *O. insidiosus.* However, in all exclusion cages, predators that should have been excluded were sometimes present. This occurred because adult insects (particularly Coccinellidae, Chrysopidae, and Syrphidae) laid eggs on the outside of the mesh and neonate larvae crawled through. Whenever this occurred, the number of predators was recorded and they were removed from the cage.

### Aphid Infestation

Each exclusion cage was infested with 15 apterous soybean aphid nymphs < 48 h old obtained using the following procedure: alate soybean aphids were placed on excised soybean leaves in Petri dishes with moist filter paper for 48 hours. After this period, the alates were removed and the remaining nymphs were transferred using a camel's hair brush to infest the exclusion cages. This was done to assure even age of nymphs and also to mimic an alates behavior of depositing nymphs and then moving to another plant, as suggested by Liu et al. (2004). Cages were infested at three different plant growth stages: vegetative (V5), beginning bloom (R1), and beginning pod set (R3). Infestation times were selected to simulate different arrival times of migrant soybean aphids. Nymphs were dispersed among the six plants by placing them onto the top expanded trifoliates.

Data were collected at approximately seven day intervals from 28 July until 29 September. On each sample date, temperature and relative humidity inside each cage were measured at canopy height by inserting a probe (EasyView 20; Extech Instruments www.extech.com) through the Velero® before opening the cage. Number of thrips per leaf were estimated on a scale of zero to four; 0 = 0 thrips per leaf, 1 = 1–10 thrips per leaf, 2 = 11–25 thrips per leaf, 3 = 26–75 thrips per leaf and 4 = >75 thrips per leaf. Soybean aphid populations early in the season were directly counted. Once populations became large, soybean aphid numbers were estimated by sampling several leaves, averaging the number of aphids, then multiplying by the number of leaves on the plant. The method of McCornack et al.(2008), although slightly different from ours, was found to be highly correlated with whole plant soybean aphid numbers. Predatory insects were directly counted; predators that should not be present were then removed. Additionally, the height of each plant in the cage was measured and plant development was recorded using the method by Fehr et al. (1971).

### Statistical Analysis

The soybean aphid and predator counts were square root transformed (x + 1) prior to analysis to fit the model's assumptions (Snedecor and Cochran 1989). Data were analyzed using repeated measures PROC MIXED (SAS 2001) (as outlined by Littell et al. (1998)). The ANOVA was a randomized complete block split plot in space and time as outlined by Steel and Torrie (1980). Blocks represented field position, the main plot was mesh, and the subplot was infestation date. The repeated measure was sampling over time in each cage. Rep within mesh infestation was used as the denominator of *F* for testing infestation and mesh × infestation. Rep × weeks after infestation (WAI) was used as the denominator of *F* for testing WAI. All other interactions were tested using the residual. Differences between means were determined using Fisher's least significant difference test. Because of differences in the number of sampling dates between infestation times (V5, 10; R1, 8; R3, 4), two separate analyses were performed (Table 1). One analysis included all four infestations (V5, R1, R3, and uninfested control) and the first four WAI. Another analysis included three infestations (V5, R1, and uninfested control) and weeks 5– 8 WAI. Samples from dates 9 and 10 WAI were not included because only comparisons between the V5 infestation and the uninfested control were possible. For treatments that exceeded the economic threshold, time to threshold was compared using PROC MIXED. Analyses of temperature, relative humidity, and plant height were performed similar to above. However, all sample dates were used and the only treatment considered was mesh type with WAI.

**Table 1A.**
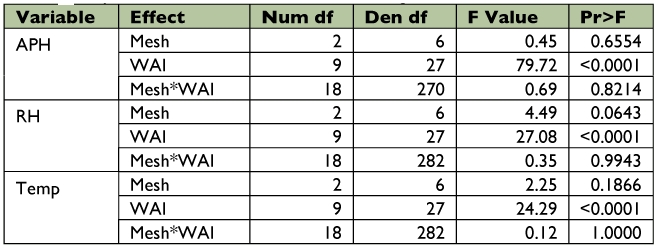
Analysis including early, middle, and late infestations and using wai 1–10.

**Table 1B.**
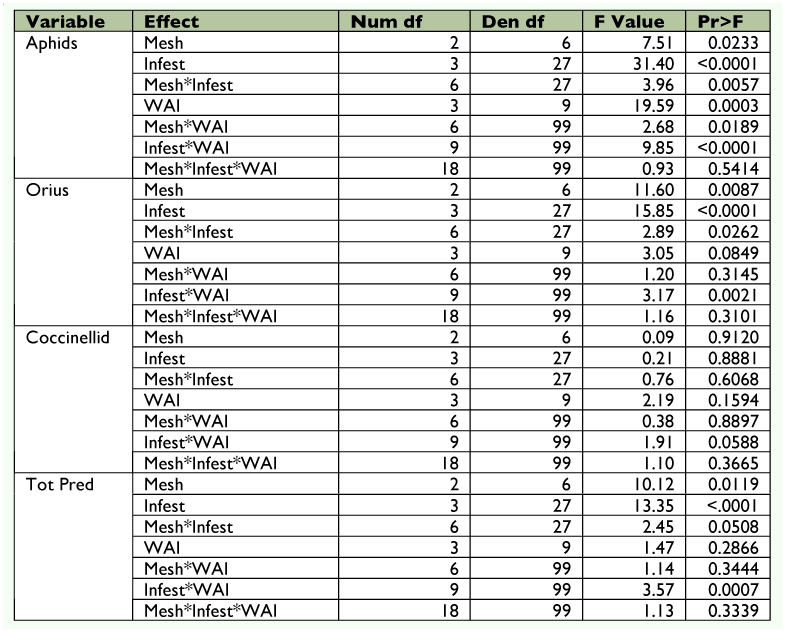
Analysis including all four infestation dates and using wai 1–4. Aphids log transformed; orius and cocc sqrt transformed.

**Table 1C.**
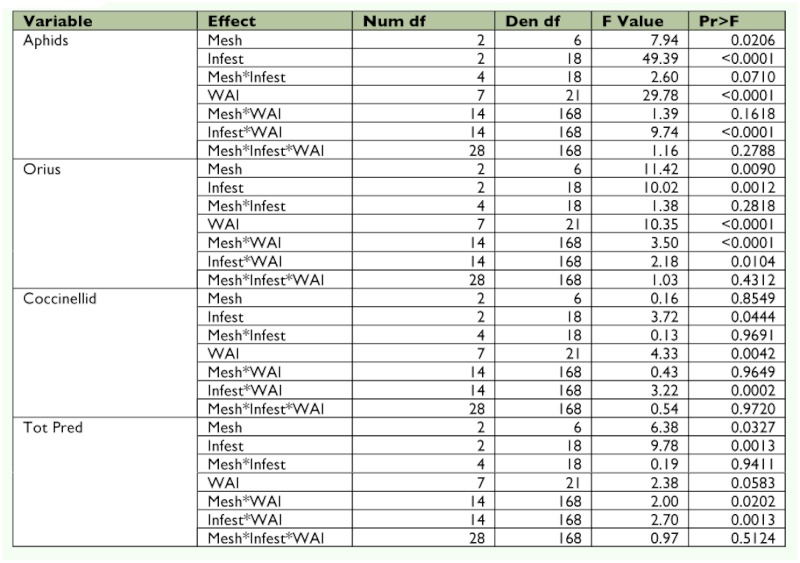
Analysis only including no, early, and middle infestations and using wai 1–8. Aphids log transformed; orius and cocc sqrt transformed.

The rate of increase of soybean aphid populations in cages of different mesh sizes was analyzed using a program created by MR Ellersieck (available on request, EllersieckM@missouri.edu). Slopes from initial infestation to peak population were determined and compared. Peak dates for V5, R1, R3, and uninfested control were 1 September, 29 September, 22 September, and 22 September, respectively. One degree of freedom polynomial contrasts were conducted in order to test differences between soybean aphid population slopes (*P* ≤ 0.05).

A stepwise regression was also performed to predict *O. insidiosus* populations as they relate to thrips populations and soybean aphid populations. As before, two separate analyses were performed. One analysis included all four infestations (V5, R1, R3, and uninfested control) and the first four WAI (Table 1). Another analysis included three infestations (V5, R1, and uninfested control) and weeks 5– 8 WAI. Sample dates 9 and 10 WAI were not included because only comparisons between the V5 infestation and uninfested control were possible. Small, medium, and large mesh treatments were included.

## Results

The rate of increase for soybean aphid populations differed significantly with treatment and infestation date (Table 2). Among cages infested at V5, aphid populations in cages with small mesh (excluding all predators) had a significantly higher (*P* ≤ 0.05) rate of increase than aphid populations in cages with medium or large mesh. Among cages infested at R1, aphid populations in cages with small and medium mesh had significantly higher (*P* ≤ 0.05) rates of increase compared to aphid populations in cages with large mesh. Cages infested at R3 and uninfested cages maintained very low populations of soybean aphid despite infestation. Uninfested cages with large and medium mesh had higher aphid populations than cages with small mesh. However, some aphids were observed in uninfested small mesh exclusion cages. Cages were 1.5 m apart and blocks were 6 m apart and all areas between cages were maintained weed free, so it is likely that stray aphids were accidently introduced by the observer from other cages.

**Table 2.**
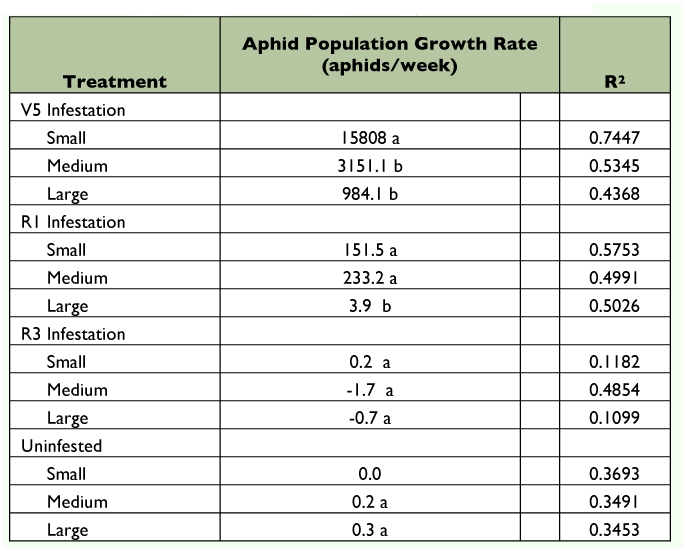
Slope and R^2^ values for A. *glycines* populations until peak during exclusion trials, 2004.

**Figure 3.**
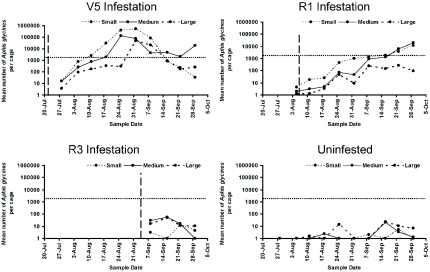
Summary of *Aphis glycines* populations by infestation date. Vertical dashed line indicates infestation date. Horizontal dotted line indicates threshold of 250 aphids per plant. High quality figures are available online.

Predator exclusion significantly affected (*P* < 0.05) the length of time from aphid infestation until economic threshold (250 aphids/plant or ∼1500 aphids/cage) was reached for the V5 and R1 infestations (Figure 3). Among cages infested at V5, economically significant populations of soybean aphid were established two, three, and four and a half weeks after infestation of small, medium and large mesh cages, respectively. Among cages infested at R1, economically significant populations of soybean aphid were established five and six weeks after infestation of small and medium mesh cages. No cages infested at R3 or uninfested cages reached the economic threshold.

**Figure 4.**
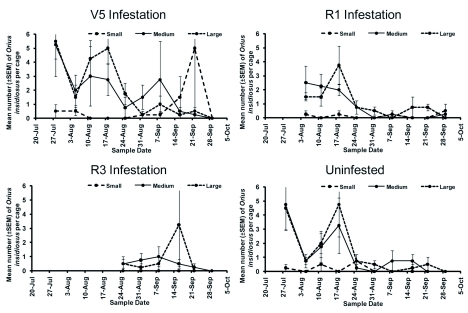
Mean number (±SEM) of *Orius insidiosus* per cage. High quality figures are available online.

**Figure 5.**
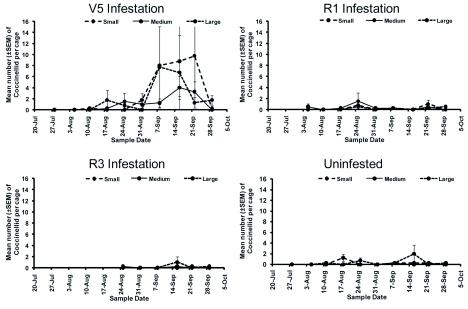
Mean number (±SEM) of coccinellids (*Coccinella septempunctata* and *Harmonia axyridis*) per cage. High quality figures are available online.

Throughout WAI 1–4, *O. insidiosus* numbers were variable and no clear pattern was discernable. In WAI 5–8, more *O. insidiosus* were found in cages infested at V5 than any other cage type (*F* = 3.89; df = 2, 28; *P* = 0.0395) (Figure 4). The most abundant predators observed during the study were *O. insidiosus* and several coccinellid species (Table 3). *Orius insidiosus* adults and immatures comprised 39.5%, while coccinellid adults and immatures comprised 37.4% of observed predators (Figure 5). *Harmonia axyridis* (Pallas) was the most prevalent coccinellid species observed, whereas *Coccinella septempunctata* (L.) was observed rarely. Syrphidae adults and immatures (9.6%) and Chrysopidae adults and immatures (4.2%) were also observed, but to a lesser extent.

During WAI 1–4, thrips numbers were a better predictor of *O. insidiosus* numbers than soybean aphid numbers (*O. insidiosus* =1.15 + 0.378 × thrips; R^2^ = 0.2185). In WAI 5–8, both thrips and soybean aphid numbers were important in predicting the number of *O. insidiosus* (*O. insidiosus* = 1.25 + 0.244 × thrips -0.049 × aphids; R^2^= 0.1781).

### Cage Effects

**Figure 6.**
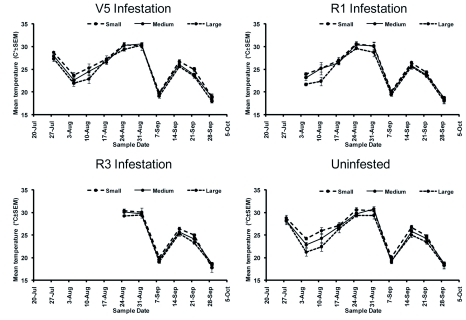
Mean temperature (±SEM) in exclusion cages. High quality figures are available online.

**Figure 7.**
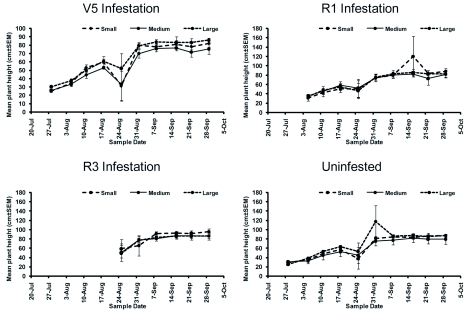
Mean plant height (±SEM) in exclusion cages. High quality figures are available online.

Temperature between mesh types differed significantly over the sampling period (*F* = 24.29; df = 27, 282; *P* < 0.0001) (Table 1); mean temperature varied by ± 1.3° C on average among mesh treatments (Figure 6). Relative humidity also differed significantly throughout the sampling period (*F* = 27.08; df = 27, 282; *P* < 0.0001) (Table 1), varying among mesh treatments by ± 3.2% on average. Plant height differed significantly over the sampling period (*F* = 79.72; df = 27, 270; *P* < 0.0001; Figure 7) (Table 1).

## Discussion

Thrips were the primary food source of *O. insidiosus* before the arrival of soybean aphid in the United States (Isenhour and Marston 1981a; Isenhour and Yeargan 1981). Research by Yoo and O'Neil (2009) suggests that thrips may serve as a food source for *O. insidiosus* early in the season, before the arrival of soybean aphid, thus assuring that *O. insidiosus* is present when soybean aphid is becoming established. Our research supports this theory, as thrips numbers were a much better predictor of *O. insidiosus* numbers early in the infestation (WAI 1–4). Later, as soybean aphid became established, both aphids and thrips were important in predicting *O. insidiosus* numbers.

Both top-down (predation) and bottom-up (plant stage) effects were found to impact soybean aphid population growth; predatory insects and increasing plant maturity decreased the rate of soybean aphid population growth (Figure 3, Table 2). Similar results were found by previous researchers, validating the importance of these effects on soybean aphid population growth (Fox et al. 2004; Fox et al. 2005; Desneux et al. 2006; Costamagna and Landis 2006; Costamagna et al. 2007a; Brosius et al. 2007; Gardiner et al. 2009).

**Table 3.**
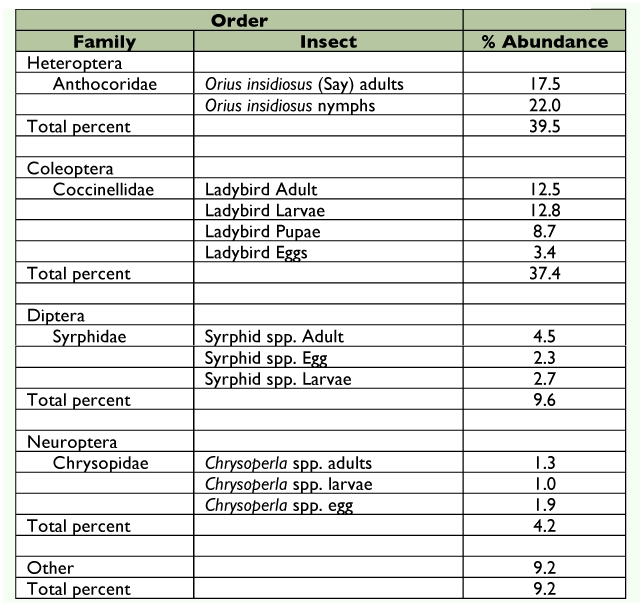
Potential *A. glycines* predators and their percent abundance during exclusion trials, 2004.

Venette and Ragsdale (2004) suggested that Missouri would provide a suitable climate for soybean aphid, but economic populations have not occurred in Missouri. However, in total predator exclusion (small mesh) cages, soybean aphid populations exceeded the economic threshold (Figure 3), suggesting that no intrinsic differences between the environments of Missouri and other Midwest states limited economic populations. Researchers such as Fox et al. (2005, 2004) and Rutledge et al. (2004) determined that predation had a significant impact on soybean aphid establishment and population growth. Our results concur with theirs and indicate that when smaller predators (mainly *O. insidiosus*) were allowed access to soybean aphid populations, aphid populations were delayed from reaching economic threshold (as in large mesh cages) (Figure 3). The role of resident predatory insects should be considered when making management decisions. Similar to other aphid species, the soybean aphid has been shown to rapidly increase population numbers following the elimination of predacious insects by insecticide application (Sun et al. 2000; Myers et al. 2005). Both *O. insidiosus* and coccinellids were present throughout the experiment and act to suppress soybean aphid population growth.

Field experiments are commonly less than perfect due to environmental uncertainties. One problem encountered during this experiment was the presence of predatory insects in cages from which they should have been excluded. This occurred because predator adults would lay eggs on the outside of the mesh and the immature insects were able to crawl through the mesh, or adults simply entered through an unnoticed opening in the Velcro®. This was a particular problem with the coccinellids in the V5 infestation date (Figure 5) at WAI 7–9. R1, R3, and uninfested cages had very low numbers of coccinellids, as expected. There was no significant difference in the number of coccinellids between mesh types, indicating that cages were equally ‘leaky’. *Orius insidiosus* was effectively kept out of the small mesh cages; however, there was no significant difference in the number of *O. insidiosus* found between the large mesh (allow *O. insidiosus*) and medium mesh (exclude *O. insidiosus*).

In exclusion cages, Liu et al. (2004) proposed three hypotheses to explain the growth of aphid populations:

1) microclimates may differ and thus affect aphid reproduction or survival
2) cages may reduce aphid emigration
3) cages may reduce aphid mortality by excluding predators

The plant growth stages used in this experiment may have affected soybean aphid establishment, survival, and subsequent reproduction. The effect of plant phenology on soybean aphid population growth has not been studied, and studies involving other aphid species are mixed on the impact of plant maturation on aphid population growth (Williams et al. 1999; Honek and Martinkova 2004). The decreasing nutritional value of maturing plants could explain why such low aphid populations were recorded for the late (R3) infestation (Figure 3); however, since different plant phonologies weren't tested simultaneously (i.e. by different planting dates), it is impossible to rule out the possibility that seasonal effects (i.e. differences in day length or temperature) were partly responsible. The data do suggest that soybean aphids establishing late in the season are less likely to need to be controlled with insecticide applications.

Cage material characteristics may have affected soybean aphid population growth by altering the microclimate. Econet S and Econet L, used in cages with small and medium mesh, reduce available light and airflow. Econet S reduces airflow by 45% and available light by 9% while Econet L reduces airflow by 5% and available light by 16% (U.S. Global Resources). These characteristics could reduce aphid mortality due to rain and wind compared to cages with large mesh. Heavy rainfall has been shown to be an important mortality factor in other aphid species (Shull 1925; Hughes 1963; Maelzer 1977; Singh 1982; Walker et al. 1984). During the experiment, the Bradford Research and Extension Center reported only three days with rainfall greater than 2.5 cm and seven days with rainfall greater than 1.25 cm. Only three days with rainfall greater than 1.25 cm and winds greater than 48 km/hr were recorded: August 4, August 24, and August 25. Thus, the impact of rain and wind seem minimal over the time of the experiment. However, the reduction in available light may have impacted the growth rate of the caged plants, though no difference in plant height was observed (Figure 7).

The optimum temperature range for soybean aphid development is reported to be between 22 and 27° C; above 32° C developmental time increases and survival rate decreases (McCornack et al. 2004; Hirano et al. 1996). No temperatures inside any of the cages rose above 32° C and the cages with the highest temperatures also had the highest number of aphids, suggesting no negative effects of high temperature in the study. Given that there was little difference between temperature, relative humidity, and plant height between cages, it seems that cage environment had little effect on soybean aphid populations.

The soybean aphid is a competent flyer and will take flight under a wide range of environmental conditions (Zhang et al. 2008). Cages would have prevented soybean aphid emigration, potentially increasing soybean aphid populations inside cages. However, large numbers of alate aphids were not observed until late September, when plants were in R5 (beginning seed set). A similar pattern of alate production was observed by Hodgson et al. (Hodgson et al. 2005). Because this was the last sampling date, it is unlikely that reinfestation of plants by alatae affected aphid populations during the course of the study.

Soybean aphid population growth is influenced by top-down (predation) and bottom-up (plant phenology) forces. Our research confirms that the presence of predatory insects decreases the rate of soybean aphid population increase. Often, this resulted in the soybean aphid population not reaching the economic threshold. Also, soybean aphid population growth was reduced on plants in later growth stages (reproductive vs. vegetative). These results suggest that predatory insect populations should be conserved (i.e. avoid insecticide application if possible) in young soybean fields to slow soybean aphid population growth, and that soybean aphid populations establishing at later plant growth stages would not need insecticide treatments.

## Abbreviations

R1: beginning bloom;
R3: beginning pod set;
V: vegetative;
WAI: weeks after infestation
